# Distribution of the members of the Pipiens Assemblage in the sympatric area from Argentina: which is where and when?

**DOI:** 10.1590/0074-02760160148

**Published:** 2016-10-24

**Authors:** María V Cardo, Alejandra Rubio, Melania Junges, Darío Vezzani, Aníbal E Carbajo

**Affiliations:** 1 Universidad Nacional de San Martín, Laboratorio de Ecología de Enfermedades Transmitidas por Vectores (2eTV), 3iA, Buenos Aires, Argentina; 2 Consejo Nacional de Investigaciones Científicas y Técnicas, Buenos Aires, Argentina; 3 Universidad Nacional del Centro de la Provincia de Buenos Aires, Facultad de Ciencias Exactas, Instituto Multidisciplinario sobre Ecosistemas y Desarrollo Sustentable, Tandil, Argentina

**Keywords:** Culex pipiens, Culex quinquefasciatus, hybrids, environmental gradients, latitude, urbanisation

## Abstract

Given their medical and veterinary relevance, the members of the Pipiens Assemblage are a worldwide target of ecological research. The distribution of *Culex pipiens* s.s. and *Cx. quinquefasciatus* converge in Buenos Aires, Argentina, where hybrids have been detected. Each member of the assemblage exhibits a distinct eco-physiological behaviour that can affect its efficiency in pathogen transmission. Our aim was to identify the environmental drivers for the spatio-temporal distribution of each member, focusing on latitudinal and urbanisation gradients. Immatures of mosquitoes were surveyed in artificial containers found within 11 public cemeteries, raised up to the adult stage and identified by their male genitalia. The distribution of each member was associated with the environment in a Generalized Linear Model. The variable accounting for most of the heterogeneity was latitude; *Cx. quinquefasciatus* was collected more frequently at northern cemeteries, whereas *Cx. pipiens* and hybrids were more likely at the southern extreme. The urbanisation gradient was also associated with the occurrence of *Cx. quinquefasciatus* and hybrids at the high and low end, respectively. Other relevant variables were cemetery total area, the proportion with graves and the presence of plastic flowers in the containers. The spatial distribution of the members of the Pipiens Assemblage within the sympatric region in South America is driven by environmental features. The information presented herein provides essential baseline data for surveillance programs and control activities.

A species complex is defined as a highly evolutionarily related group of species which are, in consequence, hard to distinguish morphologically. The mosquitoes grouped in the complex *Culex pipiens* s.l. Linnaeus, 1758 (Diptera: Culicidae), hereby referred to as the Pipiens Assemblage following Harbach (2012), represent the most important *Culex* species from a medical and veterinary standpoint because they are vectors of multiple pathogens affecting humans, domestic and wild animals (reviewed in Vinogradova 2000). In Argentina, these mosquitoes have been recently implicated as vectors of St. Louis encephalitis virus, West Nile virus and dirofilariasis (Rubio et al. 2011, Vezzani et al. 2011). Since there is no treatment or vaccine against most of these diseases, entomological surveillance and mosquito control remain as the main public health strategies.

The Pipiens Assemblage is present in almost all inhabited regions throughout the world, in intimate association with human settlements (Farajollahi et al. 2011). Among its members, two are abundant worldwide; *Cx. pipiens* s.s. (with two biotypes, *pipiens* and *molestus*) is widely distributed in temperate areas from Europe, Africa, Asia, North and South America and Australia, whereas *Cx. quinquefasciatus* Say, 1823 is present in tropical and subtropical Africa, Americas, Southeast Asia and Australia. In sympatric areas in which the distributions of both converge, fertile hybrids have been detected (Almirón et al. 1995, Vinogradova 2003).

Each member of the assemblage exhibits a distinct eco-physiological behaviour that can affect its efficiency in pathogen transmission. *Culex pipiens pipiens* is mainly ornitophilic, but also feeds on mammals including humans, *Cx. pipiens molestus* is highly anthropophillic and *Cx. quinquefasciatus* varies widely according to the geographical location from 100% mammalophilic, with many meals taken on humans, to a high degree of ornithophily (Vinogradova 2003, Takken & Verhulst 2013). The suspicion that hybrids feed both on birds and humans could make them crucial as bridge vectors (Díaz-Badillo et al. 2011). To overwinter, *Cx. pipiens pipiens* diapauses in the nulliparous fertilised female stage, *Cx. pipiens molestus* remains reproductively active throughout the year and *Cx. quinquefasciatus* enters in a temperature-induced quiescence, although its behaviour in the southern limit of its distribution is unknown (Vinogradova 2000, Kothera et al. 2009). Both environmental and genetic factors may influence vector competence and impact the ability of populations to become infected and transmit virus. Hybridisation has a significant effect on vectorial capacity, as enhanced transmission of West Nile virus was measured in hybrid populations relative to one or both parental stains (Ciota et al. 2013). These differences have wide epidemiological implications, and the lack of information regarding the spatio-temporal distribution of each member has led to confusion on their relative contribution to disease propagation. It has also led to inexact generalisations of transmission dynamics. Therefore, the identification of the members of the assemblage is highly relevant as a first step to evaluate their role in disease transmission cycles. Two approaches are currently accepted, namely the morphology of the male genitalia (Sundararaman 1949) and molecular assays (reviewed in Farajollahi et al. 2011).

In Argentina, the assemblage is present throughout the country except its southernmost end. In Buenos Aires, it is the most abundant mosquito in artificial containers (Vezzani & Albicócco 2009, Rubio et al. 2011) and one of the three most abundant in premises (Vezzani et al. 2011). It has also been collected in a wide variety of larval habitats in wild recreational areas located close to the metropolitan area (Albicócco et al. 2011, Cardo et al. 2011), in which the abundance of migratory birds contribute to the risk of entry of different arboviruses to the region. The first record of hybrids was made by Brewer et al. (1987) between 30 and 32º S. In a latitudinal transect spanning 25-42º S, Almirón et al. (1995) found exclusively *Cx. quinquefasciatus* in the northern locations, mixed samples with hybrids in the centre of the country (32º 56’ S) and only *Cx. pipiens* in the southern end. More recently, de Morais et al. (2010) reported parental and hybrid specimens in La Plata City, Buenos Aires (34º 55’ S). It is evident that the members of the assemblage are sympatric in the centre of the country, hence the latitudinal strip 30-36º S has been recently postulated for the presence of hybrid populations (Diez et al. 2012).

The differential distribution of the members of the assemblage may be influenced by several factors. Seasonality plays a main role for insect communities in temperate regions; temperature, relative humidity and photoperiod partially determine the onset and duration of their life cycle and the occurrence of diapause (Edillo et al. 2009). Urbanisation has a pronounced effect on both abiotic and biotic components of the environment, changing resource availability and connectivity among optimal habitat patches, and therefore affecting the spatial pattern of species distribution (Carbajo et al. 2006). An inherent characteristic of urban areas is the high availability of waste water drainage systems and artificial containers, that when filled with rain or tap water become habitats for immature stages of mosquitoes (Vezzani 2007). It has also been described that urban development strongly modifies the spatial patterns of climatic variables, mainly temperature (Leveratto et al. 2000).

Beyond the evident latitudinal effect, the ecological determinants of the distribution of the members of the Pipiens Assemblage have not yet been studied in Argentina. The present paper aims to investigate the effect of the environment at different scales on the distribution of each member within the sympatric region in Argentina. In particular, we intend to (i) characterise the seasonal variations of the immature populations of *Cx. pipiens*, *Cx. quinquefasciatus* and their hybrids; (ii) evaluate the distribution of each member of the assemblage as a function of latitudinal and urbanisation gradients; and (iii) investigate the association of the distribution of each member with characteristics of the environment at the microscale.

## MATERIALS AND METHODS

**Fig. 1:**
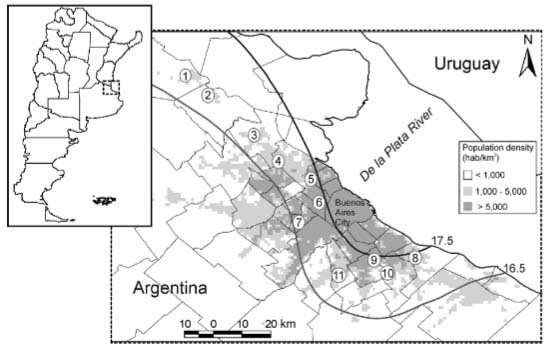
: location of the 11 cemeteries surveyed, numbered from north to south. The urbanisation density map is superimposed on the study area arranged in three categories (< 1,000, 1,000-5,000 and > 5,000 inhabitants/km2), along with isotherms marking the band 16.5-17.5ºC. Upper left: position of the study area (dotted square) in southern South America.

*Study area* - The study area extends between 34.1 and 34.9º S, within Buenos Aires Province. The climate is temperate humid-subhumid, with average temperature and cumulative precipitation of 17ºC and 1,076 mm, respectively (Cardo et al. 2014). The original grassland has been partially or totally replaced by agriculture, farming and human settlements. The area includes 11 districts which vary widely in population density (93 - 7,551 hab/km^2^) (Cardo et al. 2014), located up to 85 km away from Buenos Aires City, the capital of Argentina (Fig. 1).

*Study design* - Within a landscape in constant process of fragmentation and urbanisation, it is imperative to select study patches in which natural and anthropic factors converge and their relations can be quantified. Public cemeteries provide such integral unit of research given their broad distribution, their relative intrinsic homogeneity and, at the same time, their variable surroundings in relation to urbanisation. In cemeteries of this area, immatures of *Cx. pipiens* s.l. are the most abundant mosquitoes together with *Aedes aegypti* (Vezzani & Albicócco 2009, Rubio et al. 2013), as has been reported for most urban cemeteries worldwide (Vezzani 2007).

Eleven public cemeteries (one per district) within the study area were selected for mosquito collection, based on a urbanisation x temperature map published in Cardo et al. (2014) (Fig. 1). Briefly, the urbanisation map was built by combining road maps with demographic data. First, a 1x1 km cell grid was generated and the road layer was clipped with the grid in order to measure road length per cell. Each pixel was then assigned a given population number by weighting the total population of the district by the fraction of road length of the district located within the pixel. The temperature map was based on 11 stations within or < 200 km distant from the study area, interpolating the mean monthly values for the period 2001-2010 to obtain a surface. To rule out the confounding effect of temperature, which is influenced by latitude and the heat island effect of the city, all cemeteries were located within the band 16.5-17.5ºC. In this way, the latitudinal and urbanisation gradients could be studied at a fixed temperature range.

Google Earth images for each cemetery were registered in ArcGis 9 to calculate the total area, the proportion occupied by graves (vs. mausoleums and other edifications) and the proportion of graves shaded by trees or bushes (Table I category Cemetery description). Two estimators of the urbanisation level around each cemetery were considered: the population density obtained in Cardo et al. (2014) at two scales (1 and 16 km^2^) and the proportion of impervious area (PIA) in a circle of 1 and 3 km radius around the geometric centre of each cemetery as in Rubio et al. (2013) (Table I category Urbanisation). Pearson´s correlation coefficients between each pair of continuous variables were calculated.

**TABLE I:** Environmental variables arranged by class used to characterise the cemeteries and containers in which the members of the Pipiens Assemblage breed within the sympatric region in Buenos Aires, Argentina

Variable class	Variable name	Description	Scale	Source
Urbanisation	PIA_1_	proportion of impervious area at 1 km radius	cemetery	Rubio et al. (2013)
	PIA_3_	proportion of impervious area at 3 km radius	cemetery	Rubio et al. (2013)
	Habita1pix	population number/km^2^ - 1 pixel = 1 km^2^	cemetery	Cardo et al. (2014)
	Habita4pix	population number/km^2^ - 4 pixel buffer = 16 km^2^	cemetery	Cardo et al. (2014)
Cemetery description	area	area occupied by each cemetery (ha)	cemetery	Google Earth + ArcGis
	propgrave	proportion occupied by graves	cemetery	Google Earth + ArcGis
	proptree	proportion of graves shaded by trees or bushes	cemetery	Google Earth + ArcGis
Geographic	lat	latitude (º)	container	Field data
	lon	longitude (º)	container	Field data
	alt	altitude (m.a.s.l.)	container	Field data
Micro-environment	capacity	container capacity (mL)	container	Field data
	volume	water volume (mL)	container	Field data
	matrix	grass, bare soil, impervious	container	Field data
	type	box, cylinder, others	container	Field data
	material	cement, ceramic, plastic, glass + metal	container	Field data
	color	white, black, gray, translucid, others	container	Field data
	insolation	sun, vegetation shade, building shade	container	Field data
	DF	presence of decayed flowers (yes - no)	container	Field data
	PF	presence of plastic flowers (yes - no)	container	Field data

*Field and laboratory work* - The 11 cemeteries were visited once a month from November 2013 to April 2014, covering the abundance peak of *Cx. pipiens* s.l. in the region (Vezzani & Albicócco 2009); except for cemetery 4 in Fig. 1 in which sampling was started in December due to delayed access authorisation. In addition, a subsample of six cemeteries (numbers 1, 4, 6, 8, 9 and 11) covering the entire latitudinal and urbanisation gradients was visited monthly between May and August 2014 to include winter data.

In each visit, 50 to 110 water-holding flower vases were inspected. These were selected in five groups of 10 to 22 consecutive containers, starting at five points previously randomly drawn on the cemetery map, alternating right and left rows of graves to account for a potential incidence of shade. The content of each container was emptied in a white plastic tray (31 x 26 x 5 cm) and inspected for larvae 3-4 and pupae of *Culex* spp., which were collected with a 3 mL plastic pipette or by filtering the content through a fine mesh strainer. These specimens are easily distinguishable from immatures of *Ae. aegypti* and of other Dipteran families common in artificial containers (mostly Chironomidae, Psychodidae and Muscidae). For each positive container, the characteristics detailed in Table I (categories Geographic and Microenvironment) were recorded.

The specimens were transported to the laboratory and raised until the adult stage. Females were discarded and males were individually processed for genitalia studies. Even though in artificial containers of the study area > 96% of *Culex* spp. belong to the Pipiens Assemblage (Rubio et al. 2011), the correspondence of each specimen to *Cx. pipiens* s.l. was checked by the occurrence of eight elements in the gonocoxopodite (Forattini 2002). Then, the DV/D ratio was used to identify each member; this measurement compares the extension of the ventral arms of the phallosome laterally of the dorsal arms (DV_left_ and DV_right_, which were averaged), to the distance between the dorsal arms (D). The identification followed the criterion DV/D ≤ 0,2 for *Cx. pipiens* s.s., DV/D ^3^ 0,4 for *Cx. quinquefasciatus* and 0,2 < DV/D < 0,4 for hybrids (Sundararaman 1949).

Due to the large number of specimens recovered in some of the samples, and given the fact that identification based on the male genitalia is time consuming, not all the individuals could be processed. We identified up to six males (as available) from each positive container. This decision was made after calculating that if the three members bred in the same container in equal proportions, processing six individuals would render 74.1% chance of capturing all of them, 25.5% of identifying two members and 0.4% of capturing only one. If two of the members bred in the same container in equal proportions, the probability of capturing only one of them would be 3.1%.

*Data analysis* - The Container Index (nº of positive containers / total nº of containers with water inspected) per month for both the assemblage and each member was calculated as an estimator of relative abundance. Note that in the first case, positive containers referred to containers with larvae and/or pupae, whereas in the second case they corresponded to a subset of such containers from which males were obtained.

Generalised linear models (GLM) and their extension including random effects (GLMM) were used to analyse the occurrence of *Cx. pipiens* s.s., *Cx. quinquefasciatus* and their hybrids as a function of the environment. The presence of each member per container among all positive containers for the assemblage was modelled assuming a binomial distribution of errors and applying the logit function as link. First, all the continuous variables in Table I were graphically examined for the presence of outliers; if a given variable was highly skewed, it was removed from further analysis. Then, all variables (including continuous variables up to quadratic terms) were tested as fixed factors in univariate analyses. The explanatory variables retained in the final multivariate models were selected by a stepwise forward procedure, in which all two and three-way interactions were evaluated. To discard colinearity problems, terms were added only if the resulting variance inflation factors were ≤ 4. To account for correlations from grouped observations, once the best GLM for each member was obtained, cemetery and month were tested as a random intercept (1|CEMET and 1|MONTH) and as a random intercept plus varying slopes (MONTH|CEMET). The goodness-of-fit of the models was evaluated in terms of the Akaike information criterion (AIC), and the selected model for each response variable was the one that yielded the lowest AIC value (Zuur et al. 2009). The final model fixed parameters were bootstrapped to discard the effect of very influential observations. To assess the accuracy of the selected models, the agreement Kappa index (K), which indicates the classification improvement of the final model over chance, was calculated. Given that the predicted values are a probability, an optimisation procedure was used to decide the cut-off point for presence/absence. K was calculated for each 0.01 cut-off point between the whole range of possible values (0-1) and the point that provided the best value of K was chosen as the optimal.

All analyses were performed using the open-source software R 3.1.2, with lme4, car and boot packages (R Core Team 2015).

## RESULTS

Out of 8,304 water-holding containers inspected, 7.9% (658) harboured immatures of *Cx. pipiens* s.l.. Male specimens were obtained from 352 of such positive containers (range 1-271 individuals per container), and 1,359 were identified as *Cx. pipiens* s.s.*, Cx. quinquefasciatus* or hybrids (hereafter P, Q and H, respectively) with the following DV/D ratios: P mean 0.12 [min -0.09, max 0.20]; H 0.28 [0.21, 0.39] and Q 0.59 [0.40, 1.08]. The number of identified P and H was similar, whereas Q was twice as abundant (Table II).

**TABLE II:** Total number of identified males and containers presenting one, two or the three members (Q, P and H for *Culex quinquefasciatus*, *Cx. pipiens* s.s. and their hybrids) among all positive containers for the Pipiens Assemblage. The number of containers predicted by chance was calculated based on field data and also considering the potential 25.5% error of capturing two members when the three were present (corrected nº of containers), due to the identification of up to only six male specimens per container

	Nº of identified males			Nº of containers		
	Q	P	H	Observed	Predicted by field data	Corrected
Q	631	-	-	170		
P	-	117	-	48		
H	-	-	68	30		
PH	-	214	192	76	57.68	56.62
QH	57	-	30	18	37.29	13.41
PQ	16	14	-	6	55.10	4.47
PQH	6	6	8	4	16.05	29.5
	710	351	298	352		
	1,359					

In total, Q, P and H were identified in 198, 134 and 128 containers, respectively (Table II). P and H were found together in more containers than expected by chance, whereas the opposite was verified for the pairs PQ and QH and for the joint occurrence of the three (PQH) (c^2^_(3)_ = 120.5, p < 0.0001). When considering the potential 25.5% error of omitting one of the three members in the identification (see Materials and Methods - section Field and laboratory work), the corrected values for PQ and QH decreased and, in turn, PQH value increased (Table II). Under this hypothesis, PH did not differ from chance, PQ and QH were lower than expected and PQH was higher than expected by chance (c^2^_(3)_ = 101.9, p < 0.0001).

**Fig. 2:**
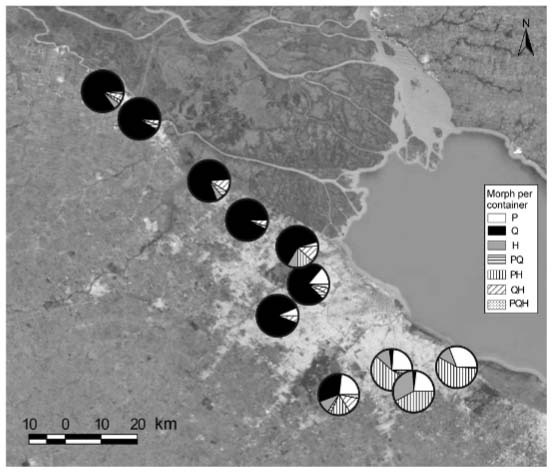
: frequency of occurrence of each morph, paired and joint coexistence per container in each of the 11 surveyed cemeteries. P, Q and H denote *Culex pipiens* s.s., *Cx. quinquefasciatus* and their hybrids, respectively.

The majority of the positive containers encountered in cemeteries 1-7 harboured only Q (Fig. 2). These cemeteries include the ones located at the northern extreme of the gradient (1-4) and the mostly urbanised (5-7). In contrast, containers harbouring only P were not recorded at northern latitudes and were present from cemetery 5 southwards. H was collected throughout the latitudinal gradient, and it was found mostly in containers at southern locations. In cemeteries 8-10, a high proportion of the positive containers presented the pair PH, in the range 0.42-0.57. As expected, the pairs PQ and QH and the joint occurrence of the three members were less abundant, although distributed all along the latitudinal gradient (Fig. 2).

**Fig. 3:**
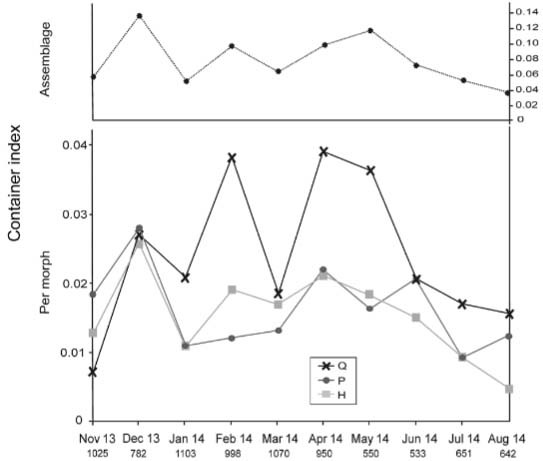
: container index (nº of positive containers / total nº of containers with water inspected) per month for the Pipiens Assemblage (upper box) and for each morph (lower box). P, Q and H denote *Culex pipiens* s.s., *Cx. quinquefasciatus* and their hybrids, respectively. Total nº of containers with water inspected is informed below each month.

*Seasonality* - The Container index for the assemblage presented values in the range 0.04 (August) - 0.14 (December), following a rough bimodal pattern with a relative peak in May (0.12) (Fig. 3, upper box).

The proportion of containers from which adults were obtained was fairly constant throughout the season [mean 0.55 (min 0.43, max 0.71)], and did not significantly correlate with the monthly Container index values of any of the members. The seasonal patterns of the three members were similar at the beginning of the season, increasing from November to December and decreasing in January (Fig. 3, lower box). Afterwards, the relative abundances of P and H were similar and lower than Q, presenting fairly stable values during the summer and early autumn with a relative peak in April, and decreasing again in winter. The relative abundance of Q presented a bell-shaped seasonal trend, although a relative minimum was registered in March.

*Environmental variables* - High pairwise correlation values were obtained among the four estimators of urbanisation (r = 0.85-0.96). The correlation coefficients of the three geographic variables with other groups was low (r < |0.6|), except for the pair cow-area (r = 0.79). As expected, the capacity and the effective water volume of each container were highly correlated (r = 0.74). No other pairwise correlations were statistically significant. The variable proptree presented an influential value in cemetery 9, therefore it was removed from further analyses.

Univariate GLMs for all environmental variables and their associations with the occurrence of the three members are presented in Table III. It is worth noticing that the behaviour of P and H for the description of the cemeteries was identical for the three variables considered and opposite to Q. Also, nearly all micro-environmental features recorded presented a distinct and opposite pattern for Q and the pair PH.

**TABLE III:** Univariate generalised linear models for the occurrence of *Culex quinquefasciatus*, *Cx. pipiens* s.s. and their hybrids. For continuous variables, the drawings represent the sign and shape of the association between each environmental variable and the lineal predictor in a binomial model. For categorical variables, the order of probability of occurrence for each level of the factor is informed

Variable class	Variable Name	*Cx. quinquefasciatus*	Hybrids	*Cx. pipiens*
Urbanisation	PIA_1_	∩	\	-
	PIA_3_	∩	\	-
	Habita1pix	-	-	-
	Habita4pix	∪	∩	∩
Cemetery description	area	\	∩	∩
	propgrave	∪	∩	∩
Geographic	lat	∩	∪	/
	lon	∩	∪	\
	alt	/	\	\
Micro-environment	capacity	∪	-	∩
	volume	-	-	-
	matrix	cement = bare soil > grass	grass > cement = bare soil	grass > cement = bare soil
	type	cylinder > box = others	ns	box = others > cylinder
	material	plastic = ceramic = glass/metal > cement	cement > plastic = ceramic = glass/metal	cement > plastic = ceramic = glass/metal
	color	black = translucid = others > white = grey	ns	white = grey > black = translucid = others
	insolation	ns	ns	ns
	DF	yes > no	ns	ns
	PF	no > yes	yes > no	yes > no

alt: altitude; lat: latitude; lon: longitude; propgrave: proportion occupied by graves; DF: decayed flowers; PF: presence of plastic flowers; PIA: proportion of impervious área; Positive linear association: /; Negative linear association: \ ; No association: - ; Quadratic association (x^2^ > 0): ∪; Quadratic association (x^2^ < 0): ∩; ns.: not significant.

*Multivariate analysis* - Null models for Q, P and H presented fairly similar AIC values (Table IV). This means that the amount of variability in the data was qualitatively equivalent for the three members.

**TABLE IV:** Best generalised linear models for the occurrence of *Culex quinquefasciatus* (Q1 and Q2), *Cx. pipiens* s.s. (P) and their hybrids (H1 and H2). The variables included as fixed factors along with the signs of the estimated slopes in the lineal predictor, the random factor, Akaike information criterion (AIC) values for the null and final model, and Kappa values with their corresponding cut-off points are informed in each case

	Fixed factors	Random factor	AIC null	AIC model	Kappa value [cut-off point]
Q1	lat - lat^2^ - area - PF	Month	484.5	179.4	0.84 [0.47]
Q2	PIA_1_ - area - PF	Cemetery	484.5	197.1	0.79 [0.31]
P	-lat + area	Month	469.7	286.4	0.73 [0.48]
H1	-lat - propgrave + PF	Cemetery	464.6	359.5	0.54 [0.43]
H2	-PIA_1_ + area - area^2^ + PF	Cemetery	464.6	359.0	0.54 [0.49]

lat: latitude; PF: presence of plastic flowers; PIA: proportion of impervious area; propgrave: proportion occupied by graves.

**Fig. 4:**
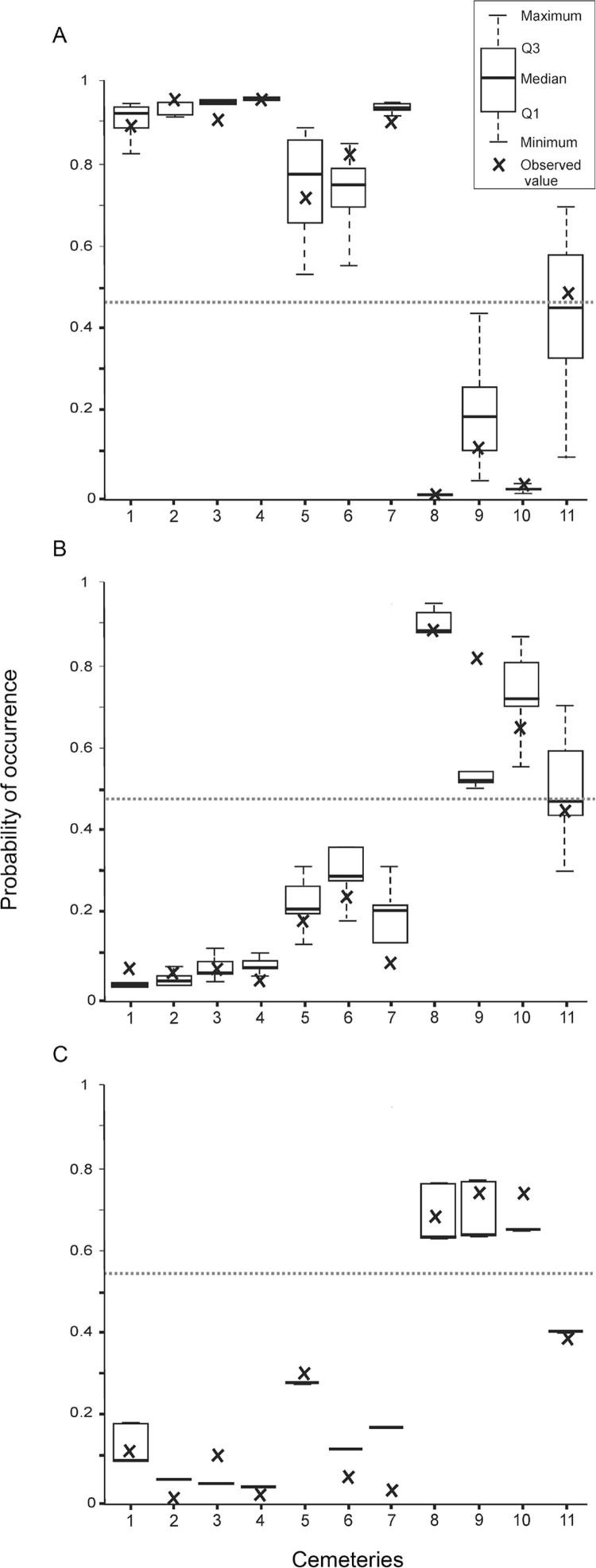
: predicted probabilities of occurrence of *Culex quinquefasciatus* (a), *Cx. pipiens* s.s. (b) and their hybrids (c) in each of the 11 cemeteries sampled, ordered from north (extreme left) to south (extreme right). The cut-off point of each model (Q1, P and H1 in Table IV) is represented by a dotted horizontal line. A prediction higher than that line indicates presence of the morph and a lower prediction indicates absence of the morph in each case. The observed proportion per cemetery is indicated by a cross.

According to the best model, the probability of occurrence of Q was higher in containers with no plastic flowers, located in small cemeteries at northern latitudes (Q1 in Table IV). The inclusion of a negative quadratic term for the latitude is explained by the fact that the cemetery located farther north presented slightly less Q than the second cemetery in the latitudinal gradient. The K value was 0.84, indicating that the occurrence of Q was 84% better predicted than by random assignment. The high quality of the model is reflected by the fact that the predicted range values per cemetery generally included the observed values (Fig. 4A boxes and crosses, respectively). The model predicted the presence of Q in cemeteries 1-7 (in which it was observed in 75-100% of all positive containers), its absence in numbers 8-10 (the three with lowest observed proportions, in the range 0-0.12) and uncertainty in number 11 (Fig. 4A).

It is noteworthy that the position on the latitudinal gradient, represented by the geographical coordinates, absorbed a high proportion of the variability in the data. When excluding these variables from the forward procedure, the urbanisation gradient emerged as significant. For instance, the model Q2 presented almost 18 more AIC points but a very good K value by including the proportion of impervious area (PIA_1_) in replacement of the latitude. The probability of occurrence of Q according to this alternative model was higher in highly urbanised, poorly shaded cemeteries (Table IV). Although at a lower cut-off point (0.31), predictions of occurrence of the members in each cemetery matched those of Q1, except for cemetery 11 in which the prediction became positive.

Regarding P, the best model predicted a higher probability of occurrence in large cemeteries located at southern latitudes (P in Table IV). The predicting capability of this model was slightly lower than for Q (73%), with P present in cemeteries 8-10 (the three with highest observed container proportions, in the range 0.65-0.89), absent in cemeteries 1-7 (observed proportions 0.02-0.23), and, as for Q, no definition in cemetery 11 (Fig. 4B). No significant models were achieved when geographical coordinates were excluded from the forward procedure.

Finally, two roughly equivalent models in terms of AIC and K values were obtained for the occurrence of H, both of which explained the observed data 54% better than by random assignment. Both models predicted a higher probability of occurrence of H in containers with plastic flowers, the first model in cemeteries with a low proportion of graves at the southern end of the latitudinal gradient and the second in lowly urbanised, mid-sized cemeteries (H1 and H2 in Table IV, respectively). Both models predicted the occurrence of H in cemeteries 8-10 and its absence in cemeteries 1-7 and 11. For both models as well as for Q2, the optimal random factor was the cemetery instead of the sampling month, accounting for the fact that the similarity of the samples taken within a cemetery outperforms the seasonality in their distribution patterns.

It is noteworthy that the models for the three members included a limited subset of the variables tested. In particular, comparing Q1 with H1 and Q2 with H2, the fixed part of the models included mainly the same variables but in opposite directions, whereas the sign of the associations in the best model for P were similar to H, matching the general results obtained in univariate analyses. This subset of variables contained representatives of the four defined variable categories: PIA_1_ for urbanisation, area and propgrave for cemetery description, latitude for geographic and PF for micro-environment. Although no significant pairwise correlations between these variables were verified, the location of the cemeteries surrounding Buenos Aires City created a weak but evident quadratic pattern in the relation between latitude and urbanisation. Most urbanised cemeteries were located at intermediate latitudes and more rural areas extended at both ends of the latitudinal gradient (see Fig. 1). Also, a slight linear positive association was noticed between latitude and area, in which cemeteries located in the southern extreme tended to be larger than northern cemeteries. As confusion among these variables could lead to erroneous interpretations, verification subsets were made as follows.

Cemeteries were first split in two groups, lowly urbanised (PIA_1_ < 0.6, cemeteries 1, 3, 4, 8, 10 and 11) and highly urbanised (PIA_1_ > 0.6, cemeteries 2, 5-7 and 9), and the models including the variable latitude (Q1, P and H1 in Table IV) were refitted for both subsets separately. Although the estimated intercepts and slopes obviously changed, the signs of the associations and the significance of the variables were the same as the ones obtained within the complete data set, with the exception of Q1, in which the quadratic term for the variable latitude was dropped (because cemeteries 1 and 2 were included in different subsets).

Then, the six cemeteries with area < 7.5 ha (1-4, 7 and 11), which covered the entire latitudinal gradient and all sampling periods, were selected. Models were refitted for this subset and, once again, the signs of the associations and the significance for the latitude remained the same.

## DISCUSSION

To our knowledge, this is the first attempt to identify the environmental drivers for the occurrence of the members of the Pipiens Assemblage in South America. As the models considered only positive containers, conditions for the breeding of immatures of *Cx. pipiens* s.l. were guaranteed and the confounding effect of including containers that could not hold immatures of the assemblage was ruled out. In other words, the models responded to the question: given that the assemblage is present, which environmental conditions would potentially favour the presence of a given member?

As expected, the variable accounting for most of the heterogeneity in the distribution of the members was latitude. Roughly, *Cx. quinquefasciatus* was segregated from the pair *Cx. pipiens* - hybrids in a northwest to southeast transect. In the Northern Hemisphere, latitudinal constraints have been recognised back in the 1950s and have been based mainly on temperature. More recently, in California, Urbanelli et al. (1997) reported a main latitudinal cline with approximately 5% introgressed individuals within the populations from both north (mainly pure *Cx. pipiens*) and south ends (mainly pure *Cx. quinquefasciatus*) of the transect, along with a narrow reversed cline characterised by increasing frequencies of *Cx. quinquefasciatus* proceeding to the north, and stated that both clines appeared to be related to temperature gradients. Afterwards, Kothera et al. (2009) reported a wider and further south extended hybrid zone and suggested that the parental subspecies and the hybrid zone are maintained by heterosis combined with the selection of diapause in northern latitudes, which is supposed to be environmentally regulated. Taking all these into consideration, we fixed temperature within a band of < 1ºC variation to discount or minimise the effects of temperature. However, this small variation may still play an important role in the distribution limit and overlapping region of the members. Other unmeasured climatic variables correlated with the latitude (*e.g.* photoperiod) could also be of relevance in the north-south segregation of the members.

The emergence and re-emergence of mosquito-borne diseases has increasingly been associated with urban landscapes. While host composition is important for zoonotic amplification, vector production in close proximity to urban settlements is a fundamental determinant of the distribution and incidence of human cases. The urbanisation gradient, estimated as the proportion of impervious surface around each cemetery, was associated with the occurrence of both *Cx. quinquefasciatus* and hybrids in a positive and negative shape, respectively. In México, *Cx. quinquefasciatus* was also collected in urban settings whereas *Cx. pipiens* s.s. was detected in suburban and rural areas (Díaz-Badillo et al. 2011). For the members of the assemblage, the urbanisation level could be an indicator of vegetation structure, availability of different feeding sources or a differential relative offer of larval habitat types. Other *Culex* species have also shown differences in their abundances according to urban landscape characteristics (*e.g.* Gleiser & Zalazar 2010).

The extension of the cemetery was also a relevant variable for the distribution of the members, being *Cx. quinquefasciatus* more frequently collected in small, *Cx. pipiens* in large and hybrids in mid-sized cemeteries. However, this variable was highly correlated with longitude, with larger cemeteries located towards the eastern extreme of the study area. Due to the configuration of the City and the selection of the study points, especially at southern latitudes, the cemeteries located towards the east are closer to the De la Plata River (see Fig. 1). This could reflect an unmeasured humidity gradient that may influence the distribution of the members.

Finally, the presence of natural or plastic flowers in a container provides shade but only the former supplies organic matter when they decay (Vezzani 2007). The probability of collecting hybrids was higher in containers with plastic flowers; on the contrary, *Cx. quinquefasciatus* was more frequently collected in containers with decayed flowers, in concordance with its common association with water with high content of organic components (Pires & Gleiser 2010).

Knowledge on vector temporal dynamics is a pre-requisite to further address numerous relevant questions in the field of epidemiology. In temperate regions, seasonal variations in climatic and ecological features such as day length, rainfall, temperature or available resources are particularly marked. We reported variations in the relative abundance of the three members along the season. However, these patterns tend to be very local, with a stochastic component and to differ among years. Given this instability, seasonality was included in the random part of the statistical modelling. This was done to account for the fact that samples taken during the same month were more alike, but avoiding to consider the season as an explanatory variable.

Regarding overwintering, with the available data it was not possible to know whether *Cx. quinquefasciatus* enters quiescence or breeds all year round in the southern limit of its distribution. Such assessment would need immature rearing from field-collected egg rafts all-year round. Although larvae were systematically collected during the winter, they could have hatched during the favourable season and stopped their development due to the low temperatures. The same situation accounts for the presence of *Cx. pipiens*, for which the absence of diapause could indicate the presence of the *molestus* biotype, which was recently reported by Micieli et al. (2013) near the study area.

The importance of the Pipiens Assemblage in disease propagation makes clarification of taxonomic relationships essential. In Argentina, the presence of hybrids and their fertility in laboratory (and presumably field) conditions, plus genetic distance and flux data, evidence the subspecific status of both taxa (Brewer et al. 1987, Almirón et al. 1995). Morphological identification of the members by means of the male genitalia has been conducted throughout the world with good results (Ohashi et al. 2014). However, Cornel et al. (2012) reported incongruence when comparing this technique with more recent molecular methods and Díaz-Badillo et al. (2011) found that, whereas *Cx. pipiens* and *Cx. quinquefasciatus* were identified almost unambiguously, some specimens morphologically identified as hybrids were classified as either *Cx. pipiens* or *Cx. quinquefasciatus* by polymerase chain reaction (PCR) of the nuclear gene Ace2. Therefore, the relative abundance of hybrids in the present study could be slightly overestimated, with a concomitant underestimation of either of the parental members. The lack of molecular identification of the members of the Assemblage is highlighted as a major limitation of this study, and this will be the focus of our research in the near future.

The infestation levels for the assemblage were within the range of previously reported data for an urban cemetery at a similar latitude (Vezzani & Albocócco 2009) and markedly lower than the levels reported for used tires in the same study area (Rubio et al. 2011). Although cemeteries are an effective proxy for the surrounding area, surveys in other land uses are planned in the near future. Another weakness of the present study is the solely inspection of artificial containers, as immatures of *Cx. pipiens* s.l. have been previously recorded in a wide diversity of aquatic habitats in the region, including tree holes and ground pools (Albicócco et al. 2011, Cardo et al. 2011). To which extent the members of the Pipiens Assemblage differentially occupy these habitats remains an unstudied issue.

Although lacking the inferential power of controlled experiments, observational studies are invaluable for describing the distributions of natural populations and identifying possible causes. We characterised the spatio-temporal dynamics of the members of the Pipiens Assemblage, providing insight in the environmental variables potentially affecting their distribution. Further studies should be extended to the entire sympatric region, to aid in the determination of the epidemic potential of arbovirus in Argentina.
